# Invasive Fungal Rhinosinusitis With Intracranial and Orbital Involvement: A Case Report

**DOI:** 10.7759/cureus.73868

**Published:** 2024-11-17

**Authors:** Haruka Nishimura, Ryo Maruyama, Masanori Yatomi, Kiyoaki Tsukahara

**Affiliations:** 1 Otolaryngology - Head and Neck Surgery, Tokyo Medical University, Tokyo, JPN

**Keywords:** aspergillus, debridement, endoscopic sinus surgery, invasive fungal rhinosinusitis, voriconazole

## Abstract

Invasive fungal rhinosinusitis can be fatal if it spreads from the orbit to the cranium. The primary treatment involves thorough lesion debridement; however, complete removal may be challenging in cases involving intracranial extension. Here, we report a case of invasive fungal rhinosinusitis with intracranial and orbital invasion successfully managed with maximal surgical debridement and antifungal therapy. The patient was a 59-year-old man with untreated diabetes mellitus who had experienced a gradual decrease in right-eye vision over the past month. He sought medical attention at a local hospital and was referred to our hospital for further examination. Computed tomography and magnetic resonance imaging scans revealed a soft tissue mass with accompanying bone destruction extending to the tip of the right orbit. Inflammation had spread to the dura mater, and cavernous sinus invasion was also observed. A biopsy was performed under endoscopy, and invasive fungal rhinosinusitis due to Aspergillus was diagnosed. We started treatment with the antifungal agent voriconazole (VRCZ), and 12 days after the biopsy, we performed endoscopic sinus surgery for debridement, removing as much of the lesion as possible. We decided to continue conservative treatment with VRCZ for the residual lesion. Two years postoperatively, the fungal lesion has maintained a reduced size. In this case, complete removal of the lesion was challenging; however, the present case suggests that disease control is possible by removing as much of the lesion as possible and administering VRCZ.

## Introduction

There are two types of fungal sinusitis: non-invasive and invasive. Most cases are non-invasive, while invasive cases are relatively rare. Invasive fungal rhinosinusitis with orbital and intracranial involvement is highly fatal and has a poor prognosis. Although the reported survival rates vary, the overall survival rate is approximately 50% [1]. Treatment involves extensive surgical resection, including orbital exenteration and craniotomy, combined with systemic antifungal drugs. However, in some cases, complete surgical resection is not possible [2,3]. There are no clear guidelines regarding the extent of resection, but recent reports indicate that disease control can be achieved even without extensive resection when combined with antifungal therapy [4]. Additionally, this disease may cause cerebrovascular complications due to fungal infection.

We report a case of invasive fungal rhinosinusitis with orbital and intracranial involvement that was successfully managed through maximal surgical resection and antifungal therapy. We also discuss the treatment outcomes with the antifungal agent voriconazole (VRCZ) and the importance of magnetic resonance angiography (MRA) in cranial evaluation.

## Case presentation

**Figure 1:**
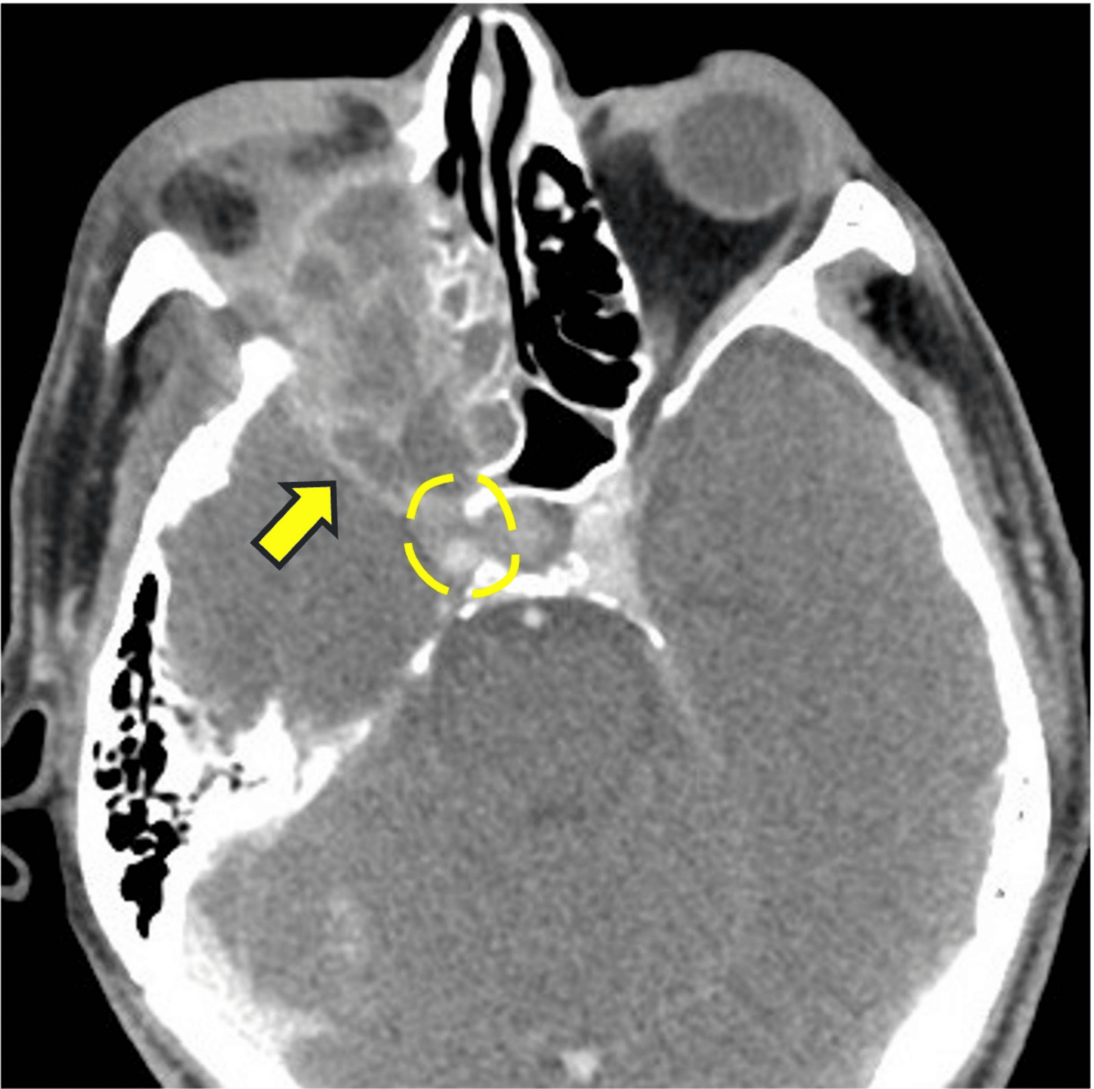
Contrast-enhanced CT of the sinus image (axial section) The lesion extended to the right orbital apex. Inflammation was also noted extending to the dura mater, with infiltration into the cavernous sinus.

The patient was a 59-year-old man with untreated diabetes mellitus who had experienced a gradual decrease in right-eye vision over the past month and sought medical attention at a local hospital. Magnetic resonance imaging (MRI) of the sinus revealed a tumor-like lesion in the right orbit. He was referred to our hospital for further examination. In the ophthalmologic examination, the right eye showed no light perception. Ocular motility disorders and diplopia could not be evaluated due to decreased vision. No ptosis or eye pain was observed. Contrast-enhanced computed tomography (CT) of the sinus (Figure 1, axial section) showed a soft tissue mass with accompanying bone destruction extending to the right orbital apex. Inflammation had spread to the dura mater, and cavernous sinus invasion was also observed.

**Figure 2:**
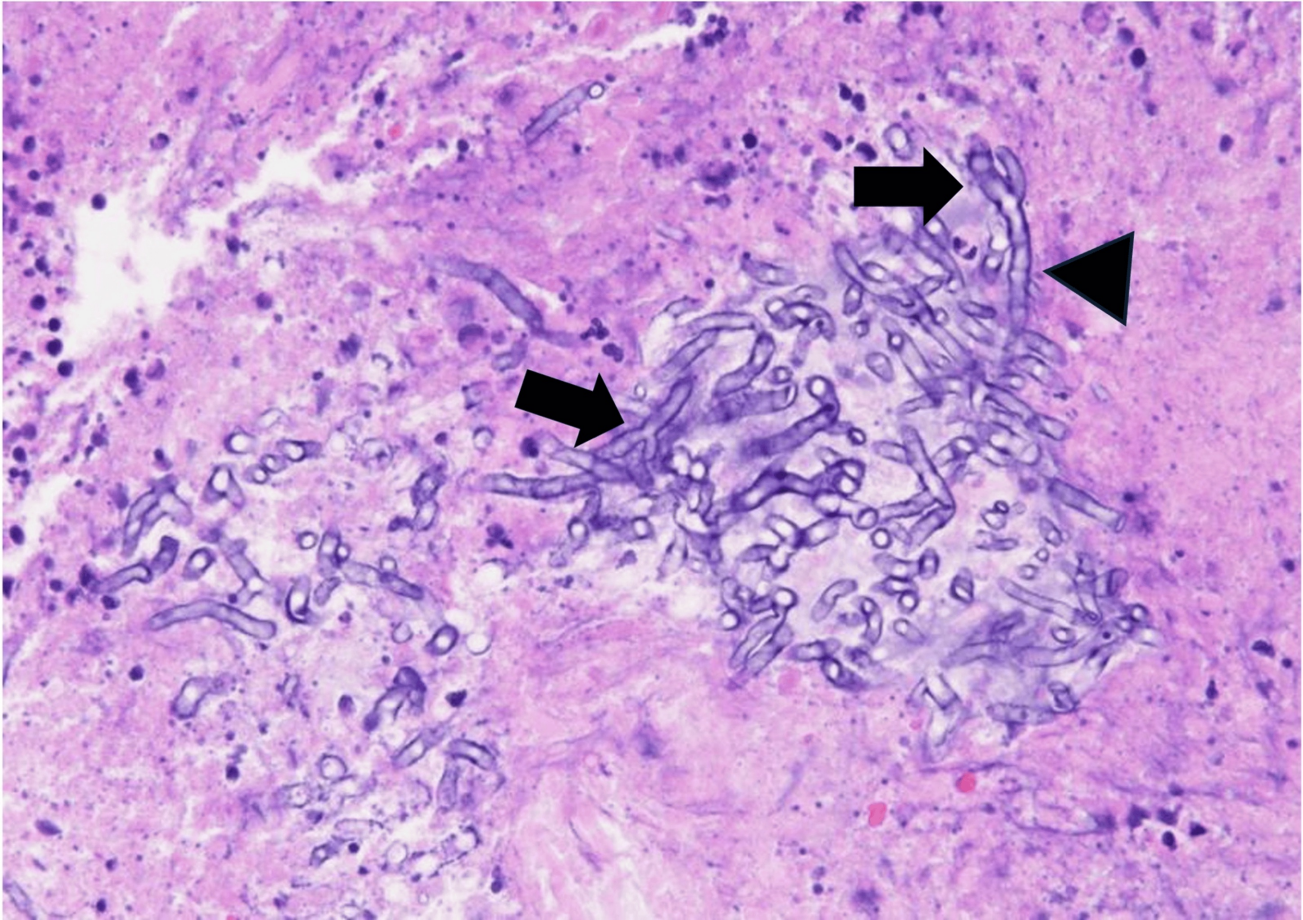
Hematoxylin and eosin stain (x400) The proliferation of fungal hyphae with 45-degree branches (arrow) and septa (arrowhead) was observed.

MRI of the sinus revealed no invasion into the brain parenchyma. Magnetic resonance angiography (MRA) showed no aneurysms or stenosis/occlusion of the cerebral vasculature. A blood test revealed a hemoglobin A1c level of 11.4%, a β-D-glucan of 28.5 pg/mL, and a squamous cell carcinoma (SCC) of 1.7 ng/mL. The patient had untreated diabetes and was admitted to the hospital for blood sugar control. The blood test results revealed elevated β-D-glucan levels and the possibility of invasive fungal rhinosinusitis was considered. However, the possibility of a malignant tumor could not be excluded. Therefore, a biopsy was performed under general anesthesia for diagnostic purposes. The biopsy showed inflammatory cell infiltration in the mucosa and the presence of fungal hyphae with 45° branches and septa, consistent with Aspergillus species (Figure 2).

**Figure 3:**
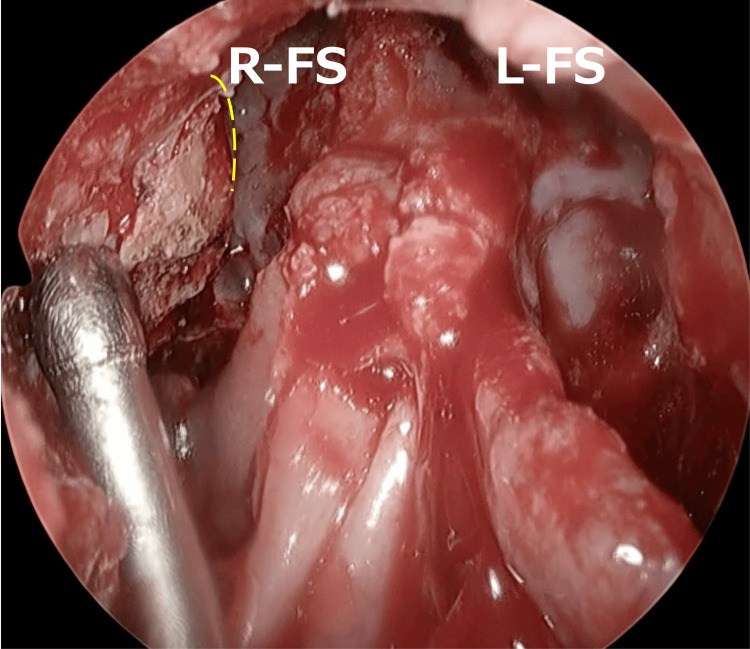
Draf type III We confirmed the upper limit of the anterior cranial base and superior wall of the orbit (dotted line). R-FS: right frontal sinus; L-FS: left frontal sinus

Based on these findings, the patient was diagnosed with invasive fungal rhinosinusitis. Intravenous VRCZ was initiated at a dose of 400 mg/day, which was gradually tapered. Although we considered performing further debridement as the lesion extended to the cavernous sinus, we thought that complete removal would be difficult. Therefore, we decided on a policy of removing as much as possible. On the 12th day after the biopsy, endoscopic sinus surgery (ESS) was performed under general anesthesia. First, we performed a Draf type III inside-out procedure and confirmed the upper limit of the anterior cranial base and superior wall of the orbit (Figure 3).

**Figure 4:**
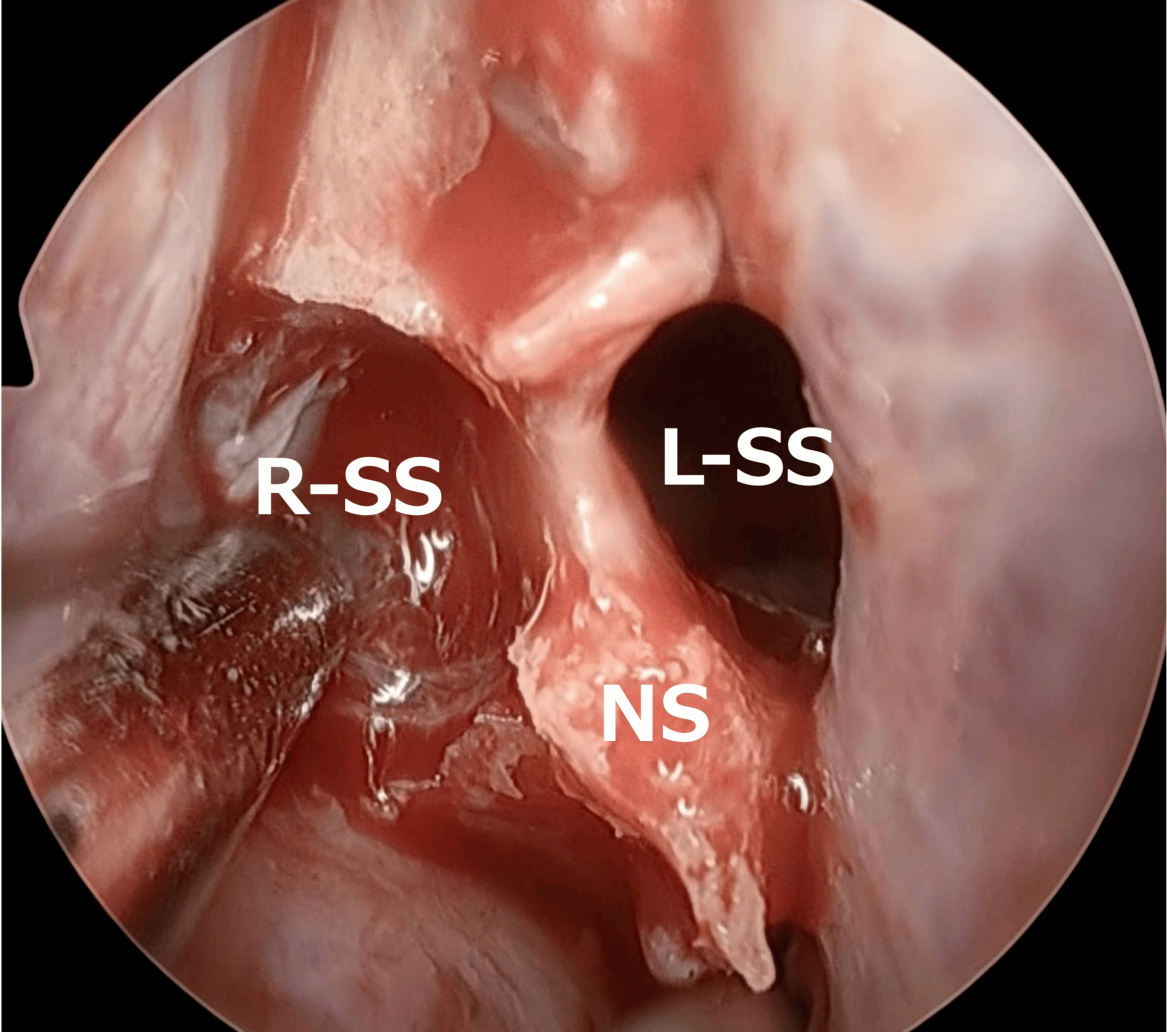
Transseptal approach An incision was made in the left nasal septal mucosa, and the left sphenoid sinus was accessed via a transseptal approach. The right sphenoid sinus was then opened laterally to the septum. R-SS: right sphenoid sinus; L-SS: left sphenoid sinus; NS: nasal septum

Next, an incision was made in the left nasal septal mucosa, and the left sphenoid sinus was accessed via a transseptal approach. The right sphenoid sinus was then opened laterally to the septum (Figure 4). 

**Figure 5:**
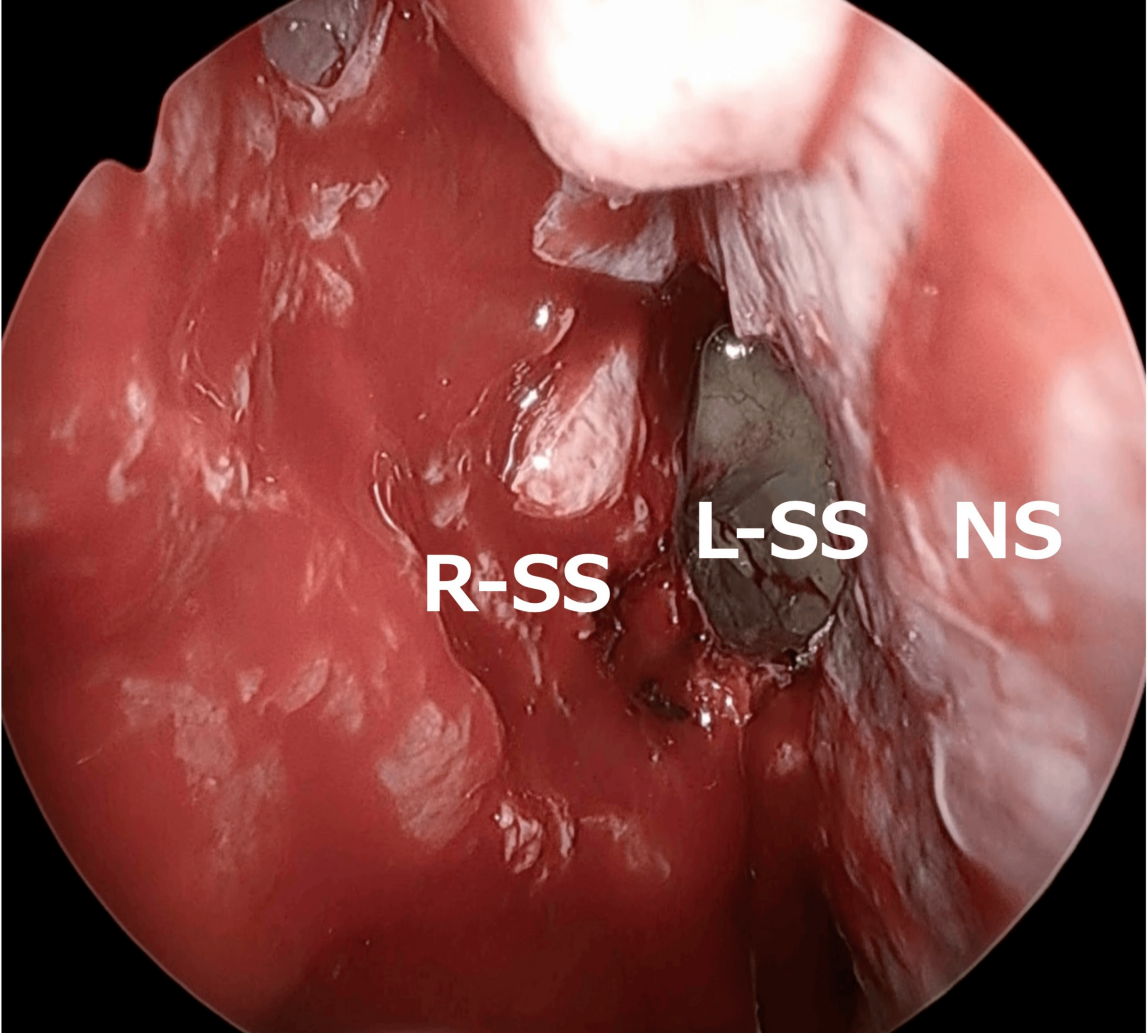
Posterior nasal septum resection The posterior part of the nasal septum was removed, and while carefully visualizing the left sphenoid sinus, the right sphenoid sinus was treated. The prominence of the internal carotid artery was identified posteriorly to the left sphenoid sinus, and this was established as the posterior limit. R-SS: right sphenoid sinus; L-SS: left sphenoid sinus; NS: nasal septum

Afterward, the posterior part of the nasal septum was removed, and while carefully visualizing the left sphenoid sinus, the right sphenoid sinus was treated. The prominence of the internal carotid artery was identified posteriorly to the left sphenoid sinus, and this was established as the posterior limit (Figure 5).

**Figure 6:**
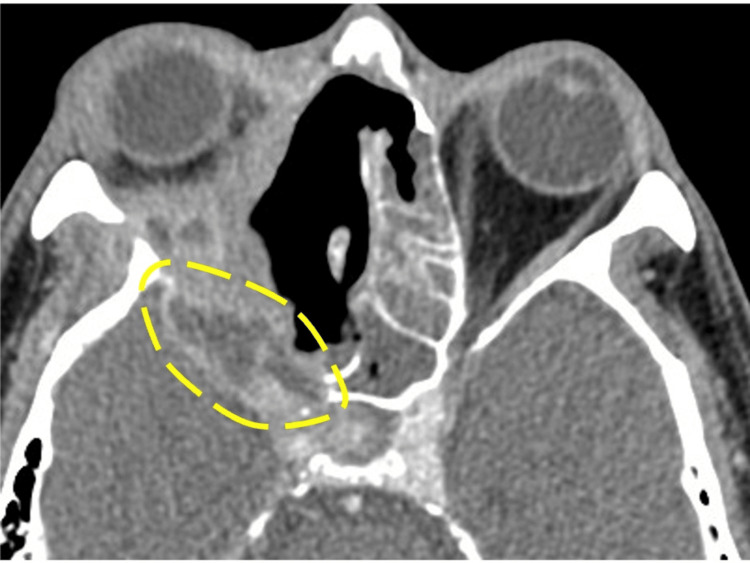
Contrast-enhanced CT image of the sinus immediately after surgery (axial section) The lesion at the tip of the orbit remained (dotted line).

Subsequently, necrotic tissue was debrided from the ethmoid sinus side toward the orbital apex. The extraocular muscles were not visualized. During the process near the orbital apex, nerve fibers were identified. Using the navigation system, these nerve fibers were confirmed to be the optic nerve, and this was established as the lateral limit. The maxillary sinus was opened through the membranous portion, and the inferior turbinate and medial wall of the maxillary sinus were resected. The anterior wall of the maxillary sinus was dissected subperiosteally from the piriform aperture, and the anterior wall of the piriform aperture and the medial part of the anterior wall of the maxillary sinus were partially resected to remove the lesion from the anterior inferior wall of the orbit. The mucosa of the left nasal septum was sutured using 5-0 PDS. The surgical area was irrigated with saline and cauterized using a suction coagulator to control bleeding, completing the surgery. The operation time was eight hours 46 min, and the blood loss was 992 mL. No intra-operative complications were observed. Immediately after surgery, contrast-enhanced CT of the sinus was performed (Figure 6, axial section), and the lesion at the right orbital apex remained.

**Figure 7:**
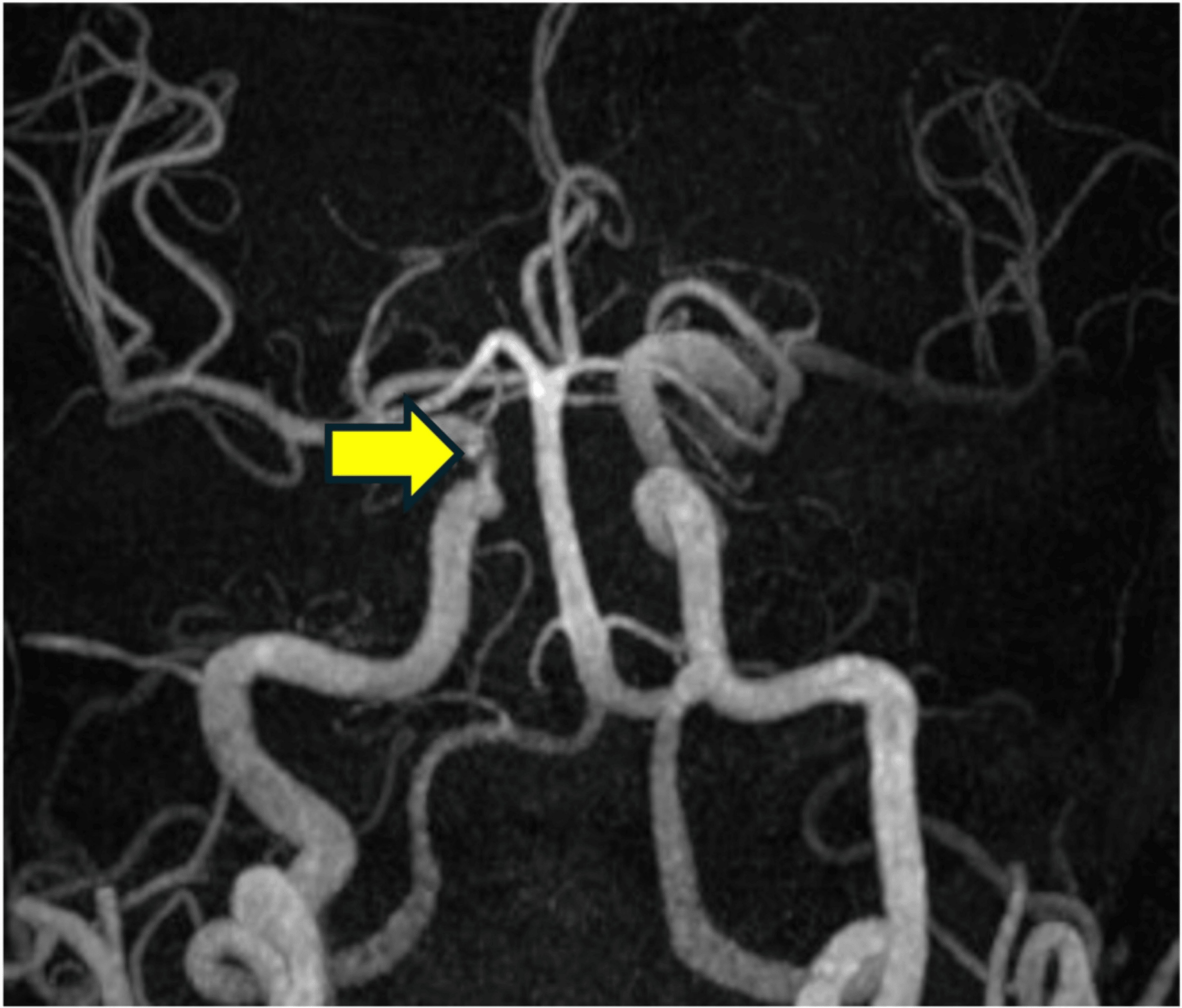
MRA 14 days after surgery Stenosis of the right internal carotid artery (arrow) was observed. MRA: magnetic resonance angiography

On the 14th day after surgery, MRA revealed a stenosis of the right internal carotid artery (Figure 7).

**Figure 8:**
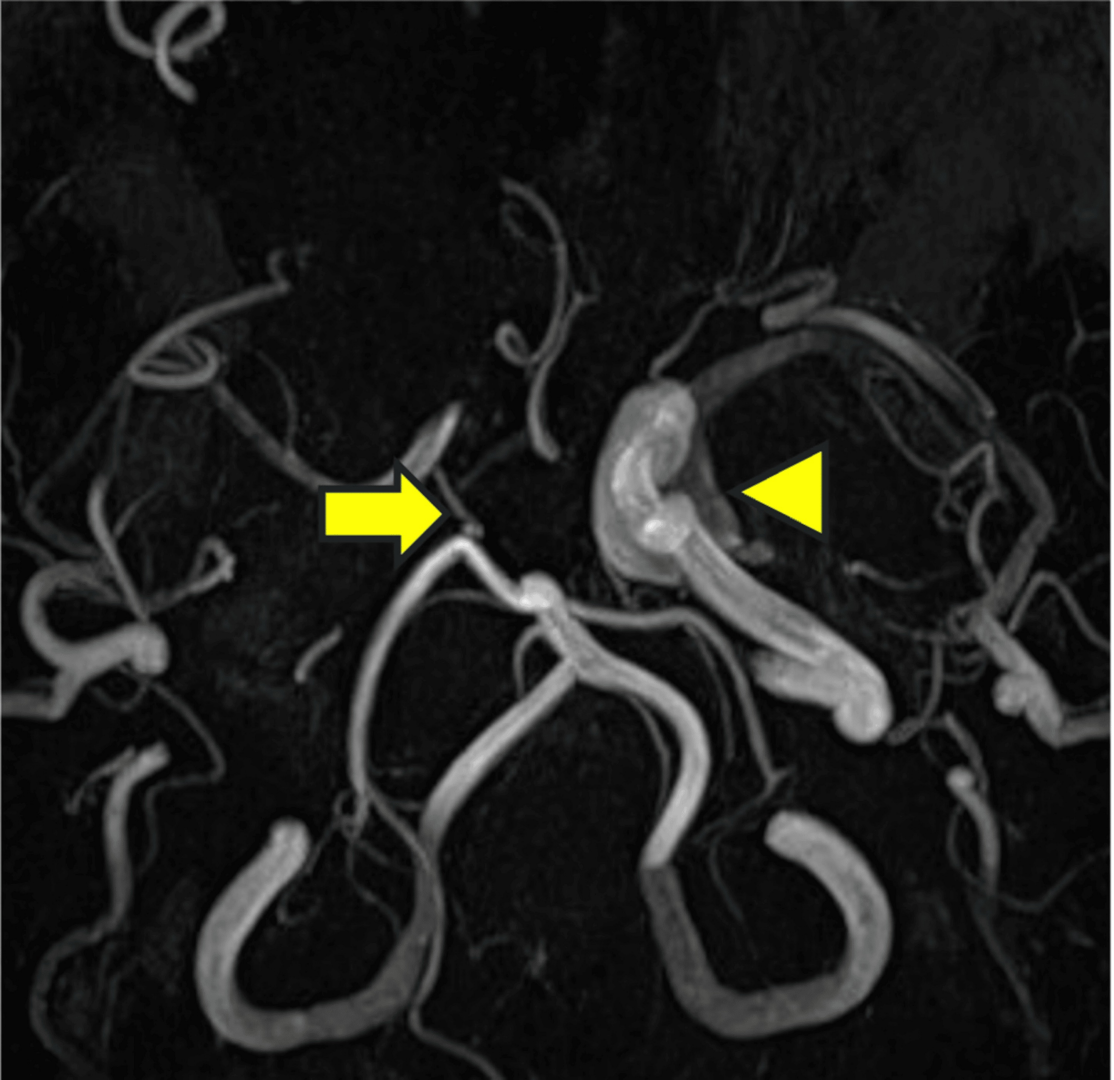
MRA one month after surgery Occlusion of the right internal carotid artery (arrow) was also observed. arrowhead: the left internal carotid artery MRA: magnetic resonance angiography

Consultation with neurosurgery led to cerebral angiography, which confirmed stenosis of the right internal carotid artery. There was a narrowing of the right internal carotid artery; however, the development of collateral blood vessels meant that the risk of cerebral infarction was low. Moreover, as the stenosis was in the cavernous sinus, the risk of a hemorrhagic subarachnoid hemorrhage was also low. Therefore, regular imaging follow-up was decided as the treatment plan. One month after the operation, MRA showed a complete occlusion of the right internal carotid artery (Figure 8); however, the patient did not develop a cerebral infarction owing to the formation of collateral blood vessels.

**Figure 9:**
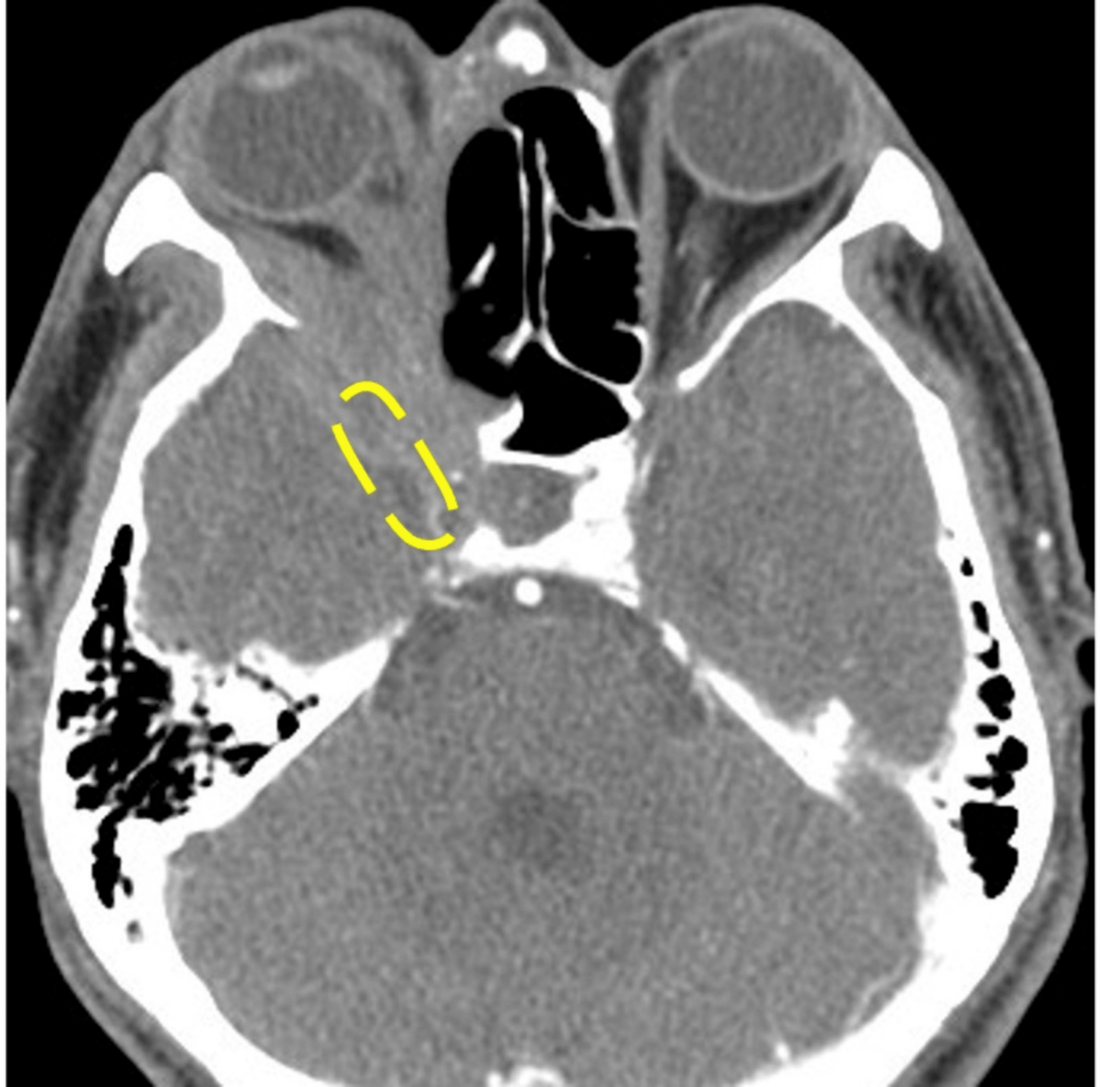
Contrast-enhanced CT of the sinus eight months after surgery (axial section) The lesion at the right orbital apex had shrunk (dotted line).

The dose of VRCZ was gradually reduced to 200 mg/day while monitoring the trough levels, and the patient was discharged 35 days postoperatively. The β-D-glucan level was 15.1 at one week postoperatively and decreased to 14.9 at two months postoperatively and then <6.0 at six months postoperatively and has remained stable since then. Contrast-enhanced CT of the sinus eight months after surgery (Figure 9, axial section) showed that the lesion at the right orbital apex had shrunk.

There were no signs of recurrence or progression of cerebrovascular disease. There has been no recovery of vision, and even after two years, the patient is still continuing oral VRCZ treatment

## Discussion

**Figure 10:**
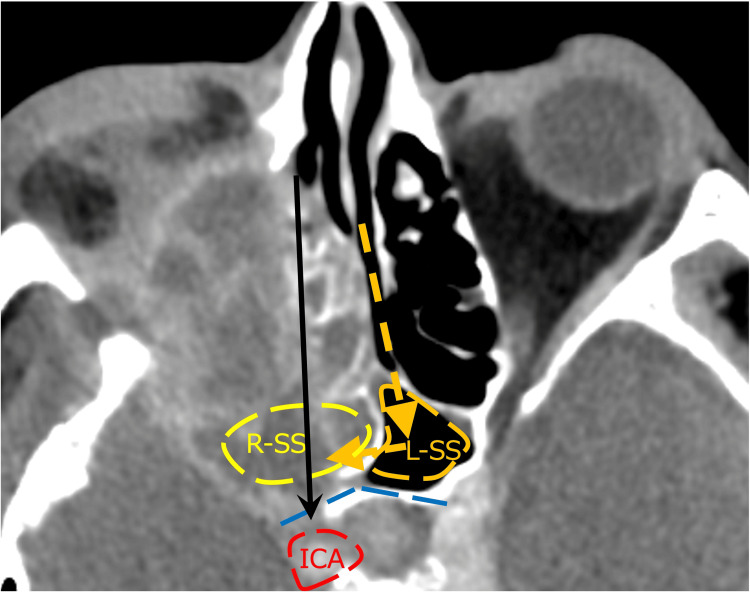
Contrast-enhanced CT of the sinus image (axial section) The right sphenoid sinus was obscured due to bone destruction. Additionally, the posterior wall of the right sphenoid sinus was destroyed, exposing the internal carotid artery. Therefore, performing debridement from the ethmoid sinus side posed a risk of internal carotid artery injury (arrow). By combining the transseptal approach with posterior nasal septum resection (dashed arrow), debridement of the ethmoid sinus side could be performed while keeping the posterior wall of the opposite sphenoid sinus (dashed line) under direct visualization, which allowed us to avoid injury to the internal carotid artery. R-SS: right sphenoid sinus; L-SS: left sphenoid sinus; ICA: internal carotid artery

There have been reports showing no difference in survival rates between groups that underwent extensive surgery, including enucleation, and those that underwent ESS for the surgical treatment of invasive fungal rhinosinusitis [1,4]. Recently, there has been an increasing number of reports favoring ESS. This shift is due to the strong bactericidal power of VRCZ and the advancement in endoscopic surgery, which has led to the reporting of various approaches and an expansion in the range of indications. In this case, we chose the ESS. Owing to the infiltration and bone destruction, the normal structures had collapsed. Therefore, we considered methods for identifying landmarks to determine the extent of resection and avoid dangerous areas before surgery. The only landmarks were the superior orbital wall, the anterior cranial base, and the contralateral sphenoid sinus. By applying Draf type III, we were able to determine the upper limit. Regarding the posterior area, the right sphenoid sinus was obscured due to bone destruction on the CT scan. Additionally, the posterior wall of the sphenoid sinus was destroyed, exposing the internal carotid artery. Therefore, performing debridement from the ethmoid sinus side posed a risk of internal carotid artery injury (Figure 10). By combining the transseptal approach with posterior nasal septum resection, debridement of the ethmoid sinus side could be performed while keeping the posterior wall of the opposite sphenoid sinus under direct visualization, which allowed us to avoid injury to the internal carotid artery. The area surrounding the internal carotid artery is a region that could be blind during an external incision, and thus ESS was considered to be useful in this case. 

Furthermore, amphotericin B (AMPH-B) was initially used as a standard treatment for invasive aspergillosis. However, the effectiveness and safety of azole antifungal agents (AAF) have been reported [5,6], and according to the 2008 Infectious Diseases Society of America guidelines, VRCZ has become the first-line treatment for central nervous system aspergillosis. Therefore, VRCZ is now the first-line treatment for invasive fungal rhinosinusitis. In 2023, Idit et al. reported the treatment outcomes of azole antifungal agents (AAF) for invasive fungal sinusitis with orbital and intracranial involvement. They retrospectively reviewed 125 cases of invasive fungal sinusitis with orbital and intracranial involvement treated with surgical intervention + AAF (VRCZ for Aspergillus, posaconazole or isavuconazole for Mucor) and compared them with 153 cases in the control group treated with surgical intervention + AMPH-B for all, with anidulafungin added in three cases and caspofungin in one case. The results showed that the AAF treatment group had a significantly lower three-year specific mortality rate compared to the control group (21% vs. 52%, respectively). The extent of the surgical procedure did not correlate with the survival rates of the AAF-treated patients. Although the AAF treatment group had more extensive intracranial involvement, 76% of cases had a high survival rate despite undergoing only conservative surgical interventions, such as without extensive procedures like cranial base surgery or enucleation. These findings suggest that disease control may be achieved through maximal removal of the lesion combined with AAF therapy, even without extensive resection. In this case, VRCZ was administered for the lesions around the dura mater and the cavernous sinus. Disease control was achieved through the most feasible removal of the lesion via ESS combined with VRCZ. Additionally, since a reduction in the residual lesions was observed over time, it is believed that VRCZ is also effective for intracranial lesions. The duration of antifungal therapy has not yet been established. In this case, disease control was achieved with a reduction in the lesion size through surgical intervention and the administration of antifungal drugs. However, complete resolution of the lesion has not been observed. Considering the patient's history of diabetes, there is concern about the potential for recurrence due to poor blood glucose control. Therefore, antifungal therapy is still being continued even after two years. Imaging evaluations are performed every six months, and if complete resolution of the lesion is observed, the discontinuation of antifungal therapy will be considered.

In addition, the involvement of the internal carotid artery suggests that Aspergillus strongly invades blood vessels. Aspergillus is known to cause arteritis and can form aneurysms through vascular invasion; however, cases of arterial occlusion are rare [7]. The occlusion mechanisms include the possibility of clot formation due to fungal cell infiltration into the wall of the internal carotid artery and the possibility of vascular lumen occlusion due to a fungal filament mass itself [8]. In this case, stenosis of the internal carotid artery was observed. However, due to the development of collateral circulation, it was judged that the risk of developing a cerebral infarction was low. Additionally, there was concern about the possibility of rupture and bleeding at the site of stenosis in the internal carotid artery. However, since the stenosis was located within the cavernous sinus, even if bleeding occurred, it would be an extradural hemorrhage, which was considered unlikely to be fatal, and it was decided to continue imaging follow-up. One month later, MRA showed occlusion of the internal carotid artery. However, no infarcted areas were observed on MRI, and since the patient was asymptomatic, it was determined that no treatment was necessary. Furthermore, as the intracranial lesions showed a trend toward reduction, observation was continued. At three months postoperatively, no further progression of cerebrovascular disease was observed, and the intracranial lesions had further reduced. Therefore, it was concluded that no further progression of cerebrovascular disease was expected. However, in cases of relapse, there is a possibility of progression of cerebrovascular lesions, so MRI and MRA evaluations are being performed every six months. In cases like this, even when no cerebrovascular abnormalities are observed preoperatively, cerebrovascular complications can be detected postoperatively. In invasive fungal sinusitis, it is important to regularly evaluate not only the sinuses but also the cerebrovascular status.

## Conclusions

We report invasive fungal rhinosinusitis with intracranial and orbital involvement. Even in cases where complete removal is difficult, the disease can be controlled by performing ESS to remove as much of the lesion as possible in combination with VRCZ therapy. In addition, since this disease can cause cerebrovascular complications, evaluating the cerebrovascular system using MRA is crucial.
